# *Salmonella enterica* subspecies *houtenae* as an opportunistic pathogen in a case of meningoencephalomyelitis and bacteriuria in a dog

**DOI:** 10.1186/s12917-020-02652-5

**Published:** 2020-11-11

**Authors:** Melissa N. Andruzzi, Mary L. Krath, Sara D. Lawhon, Beth Boudreau

**Affiliations:** 1 Department of Neurology, Texas A&M Small Animal Hospital, 408 Raymond Stotzer Pkwy, College Station, TX 77845 USA; 2 Department of Veterinary Pathobiology, Texas A&M Small Animal Hospital, 408 Raymond Stotzer Pkwy, College Station, TX 77845 USA

**Keywords:** SRMA (steroid-responsive meningitis arteritis), *Salmonella enterica* subspecies *houtenae*, Meningoencephalitis, Bacteriuria, Opportunistic infection

## Abstract

**Background:**

We report the first case of canine *Salmonella* meningoencephalomyelitis and second case of canine *Salmonella* bacteriuria, as well as the first reported case of *Salmonella enterica* subspecies *houtenae* in a dog.

**Case presentation:**

Immunosuppressive treatment in a dog for a relapse of steroid-responsive meningitis and arteritis (SRMA) allowed for the opportunistic establishment of a bacteremia with *Salmonella enterica subsp. houtenae*, ultimately causing meningoencephalomyelitis and subclinical bacteriuria. The bacterial infections were treated with a four-month course of amoxicillin; clinical treatment success was determined by serial negative urine cultures and lack of clinical signs correlated to the meningoencephalomyelitis.

**Conclusions:**

Both the bacteriuria and meningoencephalomyelitis represented opportunistic infections in a dog immunosuppressed for SRMA. The clinical course of this infectious meningoencephalitis emphasizes the importance of differentiating relapse of initial disease from opportunistic infection occurring in a compromised central nervous system. The novel *Salmonella* species identified in this case acts as a reminder that infectious disease diagnostics should not be curbed by anecdotal prediction of routine pathogenic suspects.

**Supplementary Information:**

The online version contains supplementary material available at 10.1186/s12917-020-02652-5.

## Background

The genus *Salmonella* is comprised of two species: *S. bongori* and *S. enterica*. *Salmonella enterica* is further subdivided into 6 subspecies: *enterica* (I), *salamae* (II), *arizonae* (IIIa), *diarizonae* (IIIb), *houtenae* (IV), and *indica* (VI). Of these subspecies, subsp. *enterica* is the most common cause of disease in mammals and contains 2600 serovars that can be divided into typhoidal and non-typhoidal *Salmonella* (NTS) serovars. Most commonly, NTS infections in humans and dogs induce gastroenteritis.

*Salmonella* can be found in the fecal samples of healthy, non-diarrheic dogs. In one study conducted across 11 labs in the United States, almost 2500 fecal samples of dogs with and without diarrhea had an overall *Salmonella* prevalence of 3% (~ 50% of which were non-diarrheic) [1]. Additionally, there is marked variety of *Salmonella* spp. isolated from canine gastrointestinal flora; one study reported that 5.6% of healthy dogs were found to be *Salmonella*-positive based on fecal culture, with 35 different *Salmonella* isolates belonging to six serovars of subsp. *enterica* [2].

Subspecies *salamae*, *arizonae*, *diarizonae*, *houtenae*, and *indica* have been recovered from a wide variety of animal species, most commonly from reptiles. *Salmonella enterica subsp. houtenae* was originally isolated from a cockatiel in 1978 and has been recovered from a variety of animals including mammals, birds, reptiles, and amphibians [3–11]. Interestingly opossums were also reported to carry the organism asymptomatically in their the biliary tract [10]. Infections in veterinary patients include reports of osteomyelitis in an adult female Taylor’s cantil (*Agkistrodon bilineatus taylori*)) and ovarian bacterial granulomas in a Duvaucel’s gecko (*Hoplodactylus duvaucelii*) [12, 13]. Although infrequently isolated from humans, *S. enterica* subsp. *houtenae* has been reported in association with meningitis or brain abscesses primarily in children or immunocompromised adults [14–18].

## Case presentation

In 2015, a 1-year-old female spayed Boxer was presented to the Texas A&M Veterinary Medical Teaching Hospital for neck pain. The dog was diagnosed with SRMA on the basis of consistent MRI and CSF analysis findings paired with negative infectious disease testing. Following 6 months of steroid therapy, she achieved complete clinical resolution and steroids were discontinued. Three and a half years later, the dog was presented for lumbar pain. MRI and CT of the lumbar spine were performed, revealing no abnormalities, and CSF analysis revealed a mild to moderate neutrophilic pleocytosis (58% nondegenerate neutrophils, 31% small mononuclear cells). No infectious disease testing was performed at this time. The dog was presumptively diagnosed with relapse of SRMA and treated with steroid monotherapy (1.6 mg/kg/day). Clinical response was incomplete, so prednisone was increased (2.3 mg/kg/day) and azathioprine was added (2 mg/kg/day × 14 days, then 2 mg/kg/EOD). Clinical signs subsequently resolved.

Two months after beginning immunosuppressive treatment, the dog was presented for an acute onset of seizure activity, low head carriage, and abnormal behavior. On examination, the dog was febrile, ambulatory tetraparetic with generalized proprioceptive deficits, and diffusely painful on paraspinal palpation. Quantitative aerobic culture of urine obtained via cystocentesis yielded greater than 100,000 CFU/mL of a *Salmonella enterica*. Bacterial identification was confirmed using matrix-assisted, laser desorption and time of flight (MALDI-TOF) mass spectrometry and PCR to detect the *spaQ* gene [5, 19, 20]. Brain MRI revealed multifocal T2 and FLAIR hyperintensities of the piriform and olfactory lobes with no abnormal contrast enhancement. Cerebrospinal fluid analysis showed marked neutrophilic pleocytosis (89% non-degenerate neutrophils, 10% large mononuclear cells) with rare intracellular rods, measuring approximately 1 μm × 3 μm. These bacteria were confirmed to be *Salmonella enterica* on culture of CSF [19]. Susceptibility data is provided in ‘Additional file’ in the supplemental information section. Genome sequencing of the bacterial isolates from urine and CSF identified the isolates as *Salmonella enterica subsp. houtenae* [19].

The dog’s urinary tract infection and encephalomyelitis were initially treated with a combination therapy of enrofloxacin (12.3 mg/kg/day) and trimethoprim sulfamethoxazole (13 mg/kg q12hr), as well as prednisone (0.9 mg/kg/day), levetiracetam, and analgesics. A gradual taper of azathioprine was also initiated to avoid a rebound hyperimmune response. Approximately 1 month into treatment, antibiotics were inadvertently discontinued due to marked clinical improvement and repeat urine culture performed 2 weeks later showed growth of a *Salmonella* species despite a normal neurologic exam. A three-month taper of prednisone was initiated. Amoxicillin was prescribed (13 mg/kg q8hr), resulting in a negative urine culture 3 weeks later.

Amoxicillin was again inadvertently discontinued prematurely due to clinical improvement, resulting in a positive urine culture approximately 4 months after diagnosis; amoxicillin was restarted again after this positive urine culture. Amoxicillin was then continued consistently for an additional 4 months and urine cultures at the 1-month and 4-month time points were both negative. Amoxicillin was discontinued after this 4-month negative urine culture. The dog remained free of any clinical signs during this 4-month period on amoxicillin.

Following discontinuation of antibiotics, it was the authors’ clinical discretion to not perform repeat cultures of urine, CSF, or blood, or to repeat advanced imaging unless clinical signs recurred; treating an incidental discovery of recrudescence of *Salmonella* species in urine or blood in an otherwise non-clinical and immunocompetent patient would not be a judicious practice of antibiotic stewardship, especially given the emerging concern of antibiotic resistance in *Salmonella* species.

Additionally, the dog had not had any known seizure activity since the initial seizure (noted at the time of the meningoencephalitis diagnosis), so a 6-week taper of levetiracetam was initiated once the dog was 6 months seizure-free; the dog has had no known seizure activity since.

## Discussion and conclusion

Extra-intestinal non-typhoidal *Salmonella* (NTS) infections are rare in humans. Bacteremia is reported to occur in 5–10% of humans who are infected with NTS, and immunosuppression and young age (< 1 yr) are risk factors for severe clinical outcomes [21, 22]. NTS infections of the urinary tract are uncommon. In a cohort single-center study that reviewed all cases of NTS urinary tract infections, 27% of patients had isolated symptomatic NTS urinary tract infection without gastroenteritis or *Salmonella* isolated from a fecal sample. These individuals were found to have a higher rate of underlying lower urinary tract malignancies, as well as higher rates of diabetes and underlying immunosuppressive states [23].

Here, we report the second case of *Salmonella* bacteriuria in a dog. In the previous report, a dog undergoing immunosuppressive treatment for IMPA had non-clinical bacteriuria, and *Salmonella enterica* serovar *Typhimurium* was isolated from the dog’s urine culture [24].

Central nervous system infections due to *Salmonella* spp. are uncommon in humans and have not been previously reported in dogs. In humans, structural brain disease and systemic immunocompromise have been associated with intracranial *Salmonella* infection [25, 26]. In children, meningitis is of particularly high risk in infants less than 6 months of age [22]. While meningitis is the most common presentation for *Salmonella* CNS infections, a few case reports have identified NTS as the etiologic agent for acute transverse myelitis [27, 28]. No serotype has been found to have a greater predilection for the CNS compared to others [29], although *Salmonella* Enteridis Group D1 was reported to be the most common agent in one report of *Samonella* meningitis in children [30].

The serovar isolated in this case *Salmonella enterica subsp. houtenae* is a novel serotype in dogs. This serotype is commonly identified in reptiles and amphibians and more than 90% of reptiles are asymptomatic carriers, contaminating their environment via fecal shedding [18]. In this case, geckos, lizards, and turtles are commonly found in the dog’s yard and have access to the dog’s outdoor water bowl. A limitation of this case management is the absence of cultures of various samples from the dog’s environment to support this hypothesis of source of infection. However, it is the authors’ opinion that given the 3-month time course that elapsed between initial culture diagnosis of a Salmonella species and the subsequent genome identification of *S. houtenae*, there would be low utility of post-hoc collection and culture of samples.

Of the 51 publications of clinical infection with *S. houtenae* in people, 5 are case reports of meningitis or brain abscess; 4 affected patients were children and 1 was a patient with HIV [14–18]. In all of these cases, CNS infection was recognized without any preluding or concomitant gastrointestinal signs, similar to the dog in this case report.

There is very limited evidence to guide treatment of NTS gastroenteritis, bacteremia, and complicated infections, such as meningitis and osteomyelitis. Given that NTS resides intracellularly within phagocytes after invading the gastrointestinal epithelium, antibiotics with intracellular penetration are often first line [22]. Amoxicillin/ampicillin, trimethoprim-sulfamethoxazole, fluoroquinolones, azithromycin, and third-generation cephalosporins are often recommended; aminoglycosides have poor clinical efficacy despite proven in vitro activity [22, 29]. There are also no universally-accepted guidelines for the duration for which to treat NTS infection, although a minimum of 4–6 weeks is repeatedly cited [22, 26, 29, 31]. Relapses, if they occur, are thought to most likely be recrudescence rather than re-infection, due to the intracellular persistence of NTS and difficulty achieving adequate and prolonged macrophage penetration [22].

Increasing antimicrobial resistance of *Salmonella* is an emerging concern, especially resistance to fluoroquinolones. Resistance rates varies with different serotypes; *S.* Enteritidis is reported to have shown less resistance than other serovars whereas *S.* Typhimurium definitive phage type (DT) 104 has become multidrug-resistant [21]. The authors acknowledge that retrospectively, a more judicious choice regarding initial antibiotic therapy could have been made in this particular case.

It is unclear if the lumbar pain and neutrophilic pleocytosis that occurred 3.5 years after initial SRMA diagnosis was a SRMA relapse or the first manifestation of *Salmonella* meningomyelitis, although timing suggests the former. In two studies of the clinical course of SRMA, all dogs had cervical pain on presentation, with thoracolumbar being a concurrent clinical sign in 15 and 34% of dogs; isolated lumbar pain in the absence of cervical pain was not reported in these two populations [32, 33]. Description of clinical signs associated with relapses commonly report “recurrence of previous signs,” cervical pain, lethargy, neurological deficits (paresis, ataxia), and fever [11, 33–35]. Though first relapses have been reported up to 2176 days, most occur earlier [11, 32].

This case highlights a novel opportunistic infection in an immunocompromised dog. Culture of CSF and urine as well as blood cultures should be considered in any immunocompromised animal with a compromised barrier system (e.g. dog with meningitis) presenting with potential ‘relapsing’ clinical signs attributable to their initial disease. Although *Salmonella* should be considered as a cause of opportunistic infection, we do not recommend fecal culture as a component of the diagnostic work-up given that *Salmonella* can be a found in fecal samples of healthy dogs.

## Supplementary Information

**Additional file 1.** Susceptibility data of *Salmonella* isolates. This additional file presents the susceptibility data for the *Salmonella houtenae* at the various timepoints along the dog’s clinical course, both from urine and CSF. These isolate interpretations are based upon *E. coli* breakpoints for dogs when possible and based upon human data when there are no known breakpoints.

## Data Availability

The datasets generated and/or analyzed during the current study are available in GenBank. The Genbank accession numbers are JAAAGG000000000 (TAMU36, August 2019 urine isolate), JAAAGF000000000 (TAMU38, August 2019 CSF isolate), and JAAAGE000000000 (TAMU76, October 2019 urine isolate) at BioProject accession number PRJNA600881 [https://www.ncbi.nlm.nih.gov/bioproject/PRJNA600881]. The raw sequence reads are available under the SRA accession numbers SRR10876206 [https://www.ncbi.nlm.nih.gov/sra/SRR10876206], SRR10876207 [https://www.ncbi.nlm.nih.gov/sra/SRR10876207], and SRR10876208 [https://www.ncbi.nlm.nih.gov/sra/SRR10876208].

## Abbreviations

AIDS: Acquired immunodeficiency syndrome
CNS: Central nervous system
CSF: Cerebrospinal fluid
CT: Computed tomography
FLAIR: Fluid-attenuated inversion recovery
HIV: Human immunodeficiency virus
IMPA: Immune-mediated polyarthritis
MRI: Magnetic resonance imaging
SRMA: Steroid-responsive meningitis and arteritis
T2: Transverse relaxation time
NTS: Non-typhoidal Salmonella

## References

[1] Reimschussel R, Grabenstein M, Guag J, Nemser SN, Song K, Qiu J (2017). Multilaboratory survery to evaluate Salmonella prevalence in diarrheic and nondiarrheic dogs and cats in the United States between 2012 and 2014. J Clin Microbiol.

[2] Amadi VA, Hariharan H, Arya G, Matthew-Belmar V, Nicholas-Thomas R, Pinckney R, Sharma R, Johnson R (2017). Serovars and antimicrobial resistance of non-typhoidal Salmonella isolated from non-diarrhoeic dogs in Grenada, West Indies. Vet Med Sci.

[3] Bauwens L, Vercammen F, Bertrand S, Collard J-M, Ceuster SD (2006). Isolation of Salmonella from environmental samples collected in the reptile department of Antwerp zoo using different selective methods. J Appl Microbiol.

[4] Krautwald-Junghanns ME, Stenkat JK, Iundefined S, Fundefined O, Iundefined B, Aundefined N (2013). Characterization of Salmonella isolated from captive and free-living snakes in Germany. Berl Munch Tierarztl Wochenschr.

[5] Krawiec M, Kuczkowski M, Kruszewicz A, Wieliczko A (2015). Prevalence and genetic characteristics of Salmonella in free-living birds in Poland. BMC Vet Res.

[6] Lukac M, Pedersen K, Prukner-Radovcic E (2015). Prevalence of Salmonella in captive reptiles from Croatia. J Zoo Wildl Med.

[7] Marin C, Ingresa-Capaccioni S, González-Bodi S, Marco-Jiménez F, Vega S (2013). Free-Living Turtles Are a Reservoir for Salmonella but Not for Campylobacter. PLoS One.

[8] Millan J, Aduriz G, Moreno B, Juste R, Barral M (2004). Salmonella isolates from wild birds and mammals in the Basque Country (Spain). Rev Sci Tech.

[9] Phillips WE, Hatkin JM (1978). Isolation of Salmonella houtenae from a Cockateel. Avian Dis.

[10] Runkel MS, Rodriguez LF, Moody FG, LaRocco MT, Blasdel T (1991). Salmonella infection of the biliary and intestinal tract of wild opossums. Lab Anim Sci.

[11] Zottola T, Montagnaro S, Magnapera C, Sasso S, Martino LD, Bragagnolo A (2013). Prevalence and antimicrobial susceptibility of Salmonella in European wild boar (Sus scrofa); Latium region – Italy. Comp Immunol Microbiol Infect Dis.

[12] Clancy MM, Newton AL, Sykes JM (2016). Management of Osteomyelitis Caused by Salmonella Enterica Subsp. Houtenae in a Taylor's Cantil (Agkistrodon Bilineatus Taylori) using Amikacin delivered via osmotic pump. J Zoo Wildl Med.

[13] Souëf ATL, Barry M, Brunton D, Jakob-Hoff R, Jackson B (2015). Ovariectomy as treatment for ovarian bacterial granulomas in a Duvaucels gecko (Hoplodactylus duvaucelii). N Z Vet J.

[14] Lourenço MCS, Reis EFMD, Valls R, Asensi MD, Hofer E (2004). Salmonella enterica subsp houtenae serogroup O:16 in a HIV positive patient: case report. Rev Inst Med Trop Sao Paulo.

[15] Ma JS, Chen PY, Lau YJ, Chi CS (2003). Brain abscess caused by Salmonella enterica subspecies houtenae in a patient with chronic granulomatous disease. J Microbiol Immunol Infect.

[16] Nimir AR, Ibrahim R, Ibrahim IAA (2011). Salmonella meningitis in a paediatric patient caused by *Salmonella enterica* serotype Houtenae. BMJ Case Rep.

[17] Tabarani CM, Bennett NJ, Kiska DL, Riddell SW, Botash AS, Domachowske JB (2010). Empyema of preexisting subdural hemorrhage caused by a rare Salmonella species after exposure to bearded dragons in a Foster home. J Pediatr.

[18] Wybo I, Potters D, Plaskie K, Covens L, Collard JM, Lauwers S (2004). Salmonella enterica subspecies houtenae serotype 44:z4, z23:--as a rare cause of meningitis. Acta Clin Bélg.

[19] Krath ML, Hillhouse AE, Little SV, Lawhon SD (2020). MiSeq sequencing of Salmonella enterica subsp. houtenae isolates from a dog treated for hind-limb paresis. Microbiol Resour Announc.

[20] Kurowski PB, Traub-Dargatz JL, Morley PS, Gentry-Weeks CR (2002). Detection of Salmonella spp in fecal specimens by use of real-time polymerase chain reaction assay. Am J Vet Res.

[21] Chen H-M, Wang Y, Su L-H, Chiu C-H (2013). Nontyphoid Salmonella infection: microbiology, clinical features, and antimicrobial therapy. Pediatr Neonatol.

[22] Wen SC, Best E, Nourse C (2017). Non-typhoidal Salmonella infections in children: review of literature and recommendations for management. J Paediatr Child Health.

[23] Gorelik Y, Paul M, Geffen Y, Khamaisi M (2017). Urinary tract infections due to Nontyphoidal Salmonella. Am J Med Sci.

[24] Cole SD, Palermo SM, Rankin SC (2018). *Salmonella enterica* serovar Typhimurium isolated from the urine of a dog undergoing treatment for immune-mediated polyarthritis. JMM Case Rep.

[25] Akhaddar A, Hall W, Boucetta M (2019). Subgaleal and brain abscesses due to Salmonella enteritidis following craniotomy for giant cell glioblastoma multiforme: a case report and literature review. Surg Neurol Int.

[26] Sarria JC, Vidal AM, Kimbrough RC (2000). Salmonella enteritidis brain abscess: case report and review. Clin Neurol Neurosurg.

[27] Momin RN, Rajendran N, Chong VH (2009). Transverse myelitis associated with Salmonella Nontyphi infection. South Med J.

[28] Richert ME, Hosier H, Weltz AS, Wise ES, Joshi M, Diaz JJ (2016). Acute Transverse Myelitis Associated with Salmonella Bacteremia: A Case Report. Am J Case Rep.

[29] Owusu-Ofori A, Scheld W (2003). Treatment of Salmonella meningitis: two case reports and a review of the literature. Int J Infect Dis.

[30] Wu H-M, Huang W-Y, Lee M-L, Yang AD, Chaou K-P, Hsieh L-Y (2011). Clinical features, acute complications, and outcome of Salmonella meningitis in children under one year of age in Taiwan. BMC Infect Dis.

[31] Fauth E, Freeman LM, Cornjeo L, Markovich JE, Janecko N, Weese JS (2015). Salmonella bacteriuria in a cat fed a Salmonella-contaminated diet. J Am Vet Med Assoc.

[32] Lau J, Nettifee JA, Early PJ, Mariani CL, Olby NJ, Muñana KR (2019). Clinical characteristics, breed differences, and quality of life in North American dogs with acute steroid-responsive meningitis-arteritis. J Vet Intern Med.

[33] Lowrie M, Penderis J, Mclaughlin M, Eckersall P, Anderson T (2009). Steroid responsive meningitis-arteritis: a prospective study of potential disease markers, prednisolone treatment, and long-term outcome in 20 dogs (2006-2008). J Vet Intern Med.

[34] Hilpert E, Tipold A, Meyerhoff N, Schwerdt J, Winkler S, Jurina K (2020). Steroid-responsive meningitis-arteritis in dogs in Germany: are there epidemiological or clinical factors influencing recurrence rate?. Tierärztl Prax Ausg K Kleintiere Heimtiere.

[35] Tipold A, Schatzberg SJ (2010). An update on steroid responsive meningitis-arteritis. J Small Anim Pract.

