# Supplementary material for: Salmonella enterica subspecies houtenae as an opportunistic pathogen in a case of meningoencephalomyelitis and bacteriuria in a dog
Source: BMC Vet Res. 2020 Nov 11;16:437. doi: 10.1186/s12917-020-02652-5 (PMC7659121; doi:10.1186/s12917-020-02652-5)
Supplement: Supplementary file 1 — Additional file 1. Susceptibility data of Salmonella isolates. This additional file presents the susceptibility data for the Salmonella houtenae at the various timepoints along the dog’s clinical course, both from urine and CSF. These isolate interpretations are based upon E. coli breakpoints for dogs when possible and based upon human data when there are no known breakpoints. [file 12917_2020_2652_MOESM1_ESM.docx]

**Additional file:** susceptibility data of *Salmonella* isolates

Note: These isolate interpretations are based on E. coli breakpoints for dogs when possible and humans when there are no known canine breakpoints.
